# Supplementary material for: Are carbon emissions trading and green financial instruments synergistic? -Comprehensive quantitative research based on content analysis
Source: PLoS One. 2024 Mar 7;19(3):e0298601. doi: 10.1371/journal.pone.0298601 (PMC10919601; doi:10.1371/journal.pone.0298601)
Supplement: S1 Data — (ZIP) [file pone.0298601.s001.zip › Data/地级市Z_F工作报告.zip!/║■─╧/╘└╤⌠╩╨/└┤╫╘╣1⁄2╓┌║┼í╛┬φ┐╦╩2╛▌═°í┐_2008.docx]

一、过去五年在富民强市进程中迈出了坚实步伐

市五届人大一次会议以来，全市上下在中共岳阳市委的领导下，坚持以邓小平理论和“三个代表”重要思想为指导，全面落实科学发展观，努力践行民本岳阳的执政和发展理念，“十五”计划圆满完成，“十一五”规划进展顺利。

过去的五年，是经济实力大幅提升的五年。全市生产总值由2002年的394亿元增加到2007年的895.4亿元（预计数，下同），年均增长12.3 %，人均GDP达到16768元；财政总收入由28.2亿元增加到60亿元，五年实现翻番。推进新型工业化取得重要进展。现代农业水平提升。市场消费拉力增强。县域经济发展提速，汨罗进入全省十强县（市）行列。

过去的五年，是改革开放扎实推进的五年。国有企业和国有资产管理体制改革步伐加快，农村综合改革不断深化，公共财政体系逐步建立。全民自主创业行动深入开展。招商引资和项目建设支撑经济加速发展，一批战略投资项目落地实施。口岸开放带动作用进一步增强。

过去的五年，是城乡面貌显著变化的五年。洞庭湖风光带一、二期工程、环南湖旅游走廊等一批重点项目提升了城市品位，岳阳进入国家卫生城市、国家园林城市行列。新农村建设迈出坚实步伐，交通能源建设快速推进，水利建设得到加强，生态环境逐步改善。

过去的五年，是人民群众得到更多实惠的五年。全市城镇居民人均可支配收入达到13661元，农民人均纯收入达到4288元，城乡居民储蓄存款余额增加到308亿元，家庭财产普遍增多。各项支农惠农政策落到实处。每年实施一批实事项目。

过去的五年，是社会建设全面进步的五年。各级各类教育迅速发展。公共卫生和基本医疗服务不断健全。岳阳被列为国家首批文化产业示范基地、中国楹联文化城市。人口和计划生育工作保持全省先进。

过去的五年，是民主法制建设不断加强的五年。推进有错无为问责和政务公开，更加注重科学民主决策和依法行政。社会大局和谐稳定。

二、岳阳已进入“全面提速 加快发展”的新阶段

2007年，政府工作按照市第五次党代会和市五届人大五次会议确定的目标任务，务实创新，强力推进。

注重优势优先，推动又好又快发展。工业经济实现“双百”目标，新增规模工业企业131家，年销售收入过亿元的企业增加到104家；农业产业化发展加快；岳阳楼新景区竣工、湖南旅游节开幕式成功举办提升了人气指数。全市主要经济指标创多年少有的高增幅，预计生产总值比上年增长14.5%，财政总收入增长21.6 %，城镇居民人均可支配收入增长16.5%，农民人均纯收入增长10.6%。

注重基础先行，构筑加快发展平台。基础设施加快推进，城陵矶（松阳湖）港区开工建设，交通建设从国省道到乡村公路、城乡结合部公路全面展开；岳阳文化艺术会展中心和一批城镇建设工程进展加快。产业项目强势推进，岳纸40万吨项目和一批招商引资项目开工建设，重大项目前期工作取得新进展。基础工作扎实推进，岳阳进入国家知识产权试点城市行列。

注重改善民生，办好惠民实事项目。在全省率先建立“零就业”家庭就业援助和失地农民就业长效机制；中心城区城镇低保标准提高到200元，保障水平居全省第二，县（市）区提高到160元，8.2万人纳入农村低保；290万农民参加新型农村合作医疗；23.5万人享受库区移民扶持政策；新建廉租房603套、经济适用房6.1万平方米；岳阳县城铁山引水工程竣工通水，全市新解决23万城乡居民的饮水安全问题。

注重管理创新，提升政府服务水平。突出加大政策和制度创新的力度；加大争创发展条件的力度，洞庭湖区特殊困难问题已纳入中央财政转移支付；加大优化经济环境的力度，市政府政务服务中心投入运行，社会治安综合治理考评居全省第二位。

在看到成绩的同时，我们也清醒地认识到，我市经济社会发展还面临不少困难和问题。我们一定高度重视，在今后工作中认真加以解决。

三、 力争到2013年 实现人均GDP翻两番

面对前所未有的新机遇和新挑战，面对岳阳人民过上更好生活的新期待，我们有责任、更有信心使岳阳跃上一个新的发展台阶，力争到2013年实现人均GDP翻两番。

今后五年，政府工作的基本思路是：深入贯彻党的十七大精神，以科学发展观统领经济社会发展全局，落实民本岳阳的执政和发展理念，坚持科学发展、好字优先，坚持改革开放、创新优先，坚持基础支撑、优势优先，坚持以人为本、富民优先，大力实施民本岳阳九项工程，加快实现“产业兴旺、城乡秀美、文化繁荣、民生殷实、社会和谐、走势强劲”的富民强市目标。根据市第五次党代会的总体部署，今后五年主要奋斗目标是：

——经济实力明显增强。加快转变经济发展方式，建设现代工业大市、现代物流旺市、现代旅游热市、现代农业强市取得重大进展。到2012年，全市生产总值达到1830亿元，年均增长12%以上，人均GDP达到33000元；三次产业结构调整为10:51.4:38.6，非公有制经济比重提高到55%；财政总收入达到110亿元，年均增长13%；城镇化率提高到52%，县域经济实力不断壮大。

——生态环境明显提质。建设生态文明，整体谋划洞庭湖生态修复。五年全市万元GDP能耗下降20%，主要污染物排放总量减少10%，森林覆盖率达到36.8%，营造城乡山清水秀的宜居环境，努力创建国家生态园林城市。

——人民生活明显改善。健全合理有序的收入分配格局，全市城镇居民人均可支配收入达到21000元，农民人均纯收入达到6300元，年均分别增长9%和8%，中等收入者增多，人民群众拥有更多财富。

——文化建设明显加强。覆盖全社会的公共文化服务体系逐步完善，形成区域特色文化产业，社会文化生活更加丰富多彩，人民精神风貌更加昂扬向上。

——公共服务明显拓展。社会公平正义得到更好保障，人民群众享有更多更切实的民主权利。努力使人民群众学有所教、劳有所得、病有所医、老有所养、住有所居，社会安定有序，和谐社会建设迈出坚实步伐。

四、今后五年政府工作的主要任务

今后五年，政府工作的主要任务是：

（一）推进新型工业化。突出提升层次，促进支柱产业强势发展。力争原油加工能力突破1000万吨，石化产业年销售收入突破1000亿元；力争造纸产业销售收入达到100亿元以上； “五电”并举组合开发，建设电力能源大市；力争食品加工产业销售收入达到300亿元以上。突出自主创新，培育新兴产业扩张发展。突出园区建设，引导中小企业集群发展。突出港口优势，推动沿江产业带快速发展。重点加快城陵矶（松阳湖）港区建设。高水平建设城陵矶物流园，努力把岳阳建成长江中游重要的区域物流中心；引进战略投资者临港布局大运量、大耗水工业项目，争取开辟国家级出口加工区，形成港口、物流、保税、工业“四位一体”发展的新型港口经济。

（二）推进新农村建设。加快农业产业化进程。加大支农惠农政策落实力度，把基础设施建设和社会事业发展的重点转向农村。五年改造建设通乡公路500公里、通村公路5000公里以上，到2012年全市所有乡镇和98%以上的建制村通水泥路；全面完成第三期农村电网改造，推进电气化乡村试点工作，实现城乡用电同网同价；解决78万农村人口饮水安全问题；农村沼气入户普及率达到30%以上；实现广播电视“村村通”。深化农村综合改革。提高农民创业致富能力。

（三）推进新型服务业。发展特色鲜明的旅游产业，做大支柱。依托品牌造势，使岳阳更吸引人；建好休闲基地，使岳阳能留住人；创新经营方式，使岳阳融入大旅游格局。培育持续旺盛的生活需求，拉动消费。推动商贸流通业提升水平，推动房地产业健康发展，推动社区服务业形成网络。建设开放有序的市场体系，激活要素。大力发展生产性服务业和金融保险业、中介服务业，培育“会展经济”、“楼宇经济”、“第三方物流”等新的增长点。整顿和规范市场秩序，让人们放心消费、安全消费。

（四）推进新型城镇化。努力把岳阳建成长江中游现代化工业、港口、旅游城市和水秀山清的宜居城市。加快拓城提质，增强城市吸纳带动功能。以战略眼光拉开城市主框架，做大做美中心城市。“东扩”——实施“前沿发展”，重点推进武广客站新城区建设，建设最具现代气息的新型服务和高新产业区。“北靠”——实施“沿江发展”，建设港口带动型沿江工业区。“西联”——实施“跨湖发展”，建设生态旅游和现代农产品加工区。“南延”——实施“对接发展”，重点依托大型综合旅游项目，沿洞庭湖岸线向南开发，建设休闲度假区。“中提”——实施“精品发展”，重点推进中心城区改造提质，建设宜居、商务、旅游多功能聚集区。续建洞庭湖风光带三期工程、环南湖旅游走廊和岳阳文化艺术会展中心，建设规划展览馆和洞庭湖博物馆；加快实施“城中村”和旧城改造，形成特色风貌街区；加快实施沿湖老厂区“退二进三”、“腾笼换鸟”；加强南湖、芭蕉湖、东风湖等内湖的保护和开发，着手规划恢复流水环城的生态景观。加快构建城镇体系，支撑县域经济快速发展。加快创新运作方式，提升城镇管理经营水平。努力实现由集中整治脏乱差向长效管理、塑造城市形象转变，由注重城镇建设向整体联动、促进城乡秀美转变。

（五）推进基础设施建设。构建内外通畅的综合交通运输枢纽，支持武广铁路客运专线岳阳段、岳常和随岳高速公路如期建成，争取启动京珠岳长复线和荆岳铁路建设，构建“一横三纵”高速公路网；改造升级460公里国省道干线公路，建成汨罗江、鲇鱼须、梅田湖、柳林江大桥，实现湖区干线公路“渡改桥”。建设人水和谐的现代水利体系，重点抓好湖区薄弱堤段建设、山丘区中小型水库除险加固、中型灌区续建配套以及大型排涝泵站更新改造。打造资源节约型、环境友好型的地域特色，严格守住保护耕地、改善生态、节能减排、保障民生“四条底线”。

（六）推进改革开放。坚定不移推进各项改革。加快国有企业改革和非公有制经济发展，推进经济主体多元化，以更大的决心、更硬的措施推进市属国有企业改制，毫不动摇地鼓励、支持、引导非公有制经济发展，大力推动全民创业，培育一大批有创新精神和社会责任感的企业家；加快财税体制改革，推进公共服务均等化；加快行政管理体制改革，推进行政运转高效化。全方位扩大对外开放。打破行政区划局限，全方位对接发展。积极应对长株潭城市群和武汉城市圈定为国家综合配套改革试验区的机遇和挑战，实行双向对接，力求南北逢源、乘势崛起。发挥地域优势主动对接，发挥流域优势同质共建，发挥港口口岸优势承接产业，发挥中间接点优势打开进出通道。调动一切积极因素，大力度招商引资。努力开拓国际市场，走出去参与竞争。坚持不懈优化经济环境。一切为企业基层着想，一切按公正公平执法，一切凭制度承诺问责。增强政府及各部门服务经济的意识，行政运作程序、制度安排和工作方式都要想企业所想、急基层所急，主动适应市场主体，为投资者提供优质高效服务。

（七）推进文化和社会建设。推动文化繁荣，在传承中大力培育现代工商文化，倡导形成创新创业、敢冒敢富、开放开明、守信守法的社会氛围。积极发展社区文化和企业文化，使人民群众基本文化权益得到更好保障。建设教育强市，坚持优先发展教育，巩固发展素质教育，继续保持高中阶段普职招生人数1:1，做强岳阳教育品牌。完善社会管理，切实维护社会稳定。

（八）推进改善民生。始终把实现好、维护好、发展好人民群众的根本利益作为政府一切工作的出发点和落脚点。实施扩大就业的发展战略，使更多的劳动者成为创业者。加快建立覆盖城乡的社会保障体系，让人民群众享有基本生活保障。建立基本医疗卫生制度，为人民群众提供良好的医疗卫生服务。坚持计划生育的基本国策，稳定低生育水平。让人民共享发展成果，让城乡居民特别是困难群众都能享受到公共财政的阳光。

五、扎扎实实做好2008年工作

2008年是全面贯彻落实党的十七大作出的战略部署的第一年，也是新一届政府的开局之年。全市经济社会发展预期目标是：生产总值增长12%以上，经济总量迈上1000亿元台阶。积极应对世界经济发展的新趋势，把握国家宏观调控政策的新要求，坚持好字优先、好中求快、稳中求进、紧中求活，加力加速发展，实现开局之年的新突破。

（一）抓重要规划编制。根据长株潭城市群、武汉城市圈定为国家"两型社会"综合配套改革试验区的新情况，重新审视岳阳的各项规划，在主动融合中加强规划修订和编制。重点完善全市产业发展规划、区域发展规划、民生项目专项规划，尽快形成各类规划定位清晰、功能互补、有效衔接的规划体系。

（二）抓重点工作突破。突出经济提速，一产业主要是夯实基础、加快产业化；二产业主要是优化结构、提高产业发展水平和经济效益；三产业主要是加速发展、提升层次。突出国企改制，各职能部门把改革作为头等任务，切实担负起责任。突出招商引资，进一步完善考评办法，资金到位看银行、开工建设看现场、设备水平看台帐、投产见效看税收。突出节能减排，确保完成约束性指标。

（三）抓重大项目建设。产业发展方面，加快岳纸40万吨项目和工业园区在建项目建设，争取炼化扩能技改、华能三期等年内开工；交通建设方面，建成岳荣新公路二期工程、漕溪港码头等项目，加快城陵矶（松阳湖）港区一期工程、通港路、各县（市）区干线公路等建设；城镇建设方面，完成岳阳文化艺术会展中心、青年中路等建设，加快武广客站和巴陵东路、洞庭湖风光带三期工程、县城饮水工程等建设。2008年我市将举办第二届湘商大会、中国（湖南）红色旅游文化节开幕式暨平江起义80周年纪念活动、北京奥运火炬传递等大型活动，努力办出特色、办出水平、办出实效。

（四）抓民生实事落实。面向困难群众和弱势群体，办好10件实事。对实事项目，我们一定办好办实，如期如质向各位代表交账，让人民群众满意。

六、加强政府自身建设

政府工作人员必须毫无保留地忠诚于党和人民、忠诚于事业，努力建设对人民负责、让人民满意的政府。

继续解放思想，增强工作前瞻性和创造性。坚持用党的十七大精神武装头脑，使解放思想真正体现在解决影响发展的实际问题上。始终保持昂扬向上的精神状态和工作****，以思想的不断解放推动事业的不断发展。

坚持依法行政，推进决策民主化和科学化。认真落实人大及其常委会的决议决定，定期报告重要工作；尊重和发挥人民政协的职能，广泛听取各民主党派、工商联、无党派人士和各人民团体的意见。提高政府依法管理社会的能力。把关心群众与引导群众很好地统一起来，最大限度地集中人民群众的智慧和力量，共同建设我们美好家园。

着力提高效能，强化政府执行力和公信力。加快政府管理方式创新，进一步健全行政责任体系，实行更加严格的目标管理、更加严格的绩效考核、更加严格的督查督办、更加严格的责任追究。今后凡因工作失误或失职造成公共利益受到重大损失的，都要公开道歉，勇于承担责任，并启动行政问责。

加强廉政建设，塑造公务员队伍新风貌和新形象。严格履行廉政建设责任制，把廉政的要求落实到每一个行政业务工作岗位，落实到每一项行政权力行使过程。坚决纠正损害群众利益的不正之风，坚决查处违法违纪案件。努力建设一支学习型、高素质、职业化的公务员队伍，把精力集中在想干事上，把本领体现在会干事上，把目标锁定在干成事上，全心全意为人民服务。
